# Seasonal Variations of Arctic Low‐Level Clouds and Its Linkage to Sea Ice Seasonal Variations

**DOI:** 10.1029/2019JD031014

**Published:** 2019-11-21

**Authors:** Yueyue Yu, Patrick C. Taylor, Ming Cai

**Affiliations:** ^1^ Key Laboratory of Meteorological Disaster, Ministry of Education (KLME)/Joint International Research Laboratory of Climate and Environment Change (ILCEC)/Collaborative Innovation Center on Forecast and Evaluation of Meteorological Disasters (CIC‐FEMD)/NUIST‐UoR International Research Institute Nanjing University of Information Science and Technology Nanjing China; ^2^ Climate Science Branch NASA Langley Research Center Hampton VA USA; ^3^ Department of Earth, Ocean & Atmospheric Sciences Florida State University Tallahassee FL USA

**Keywords:** Arctic cloud, seasonal cycle, cloud‐sea ice interactions, atmosphere‐surface interactions

## Abstract

Using CALIPSO‐CloudSat‐Clouds and the Earth's Radiant Energy System‐Moderate Resolution Imaging Spectrometer data set, this study documents the seasonal variation of sea ice, cloud, and atmospheric properties in the Arctic (70°N–82°N) for 2007–2010. A surface‐type stratification—consisting *Permanent Ocean*, *Land*, *Permanent Ice*, and *Transient Sea Ice*—is used to investigate the influence of surface type on low‐level Arctic cloud liquid water path (LWP) seasonality. The results show significant variations in the Arctic low‐level cloud LWP by surface type linked to differences in thermodynamic state. Subdividing the *Transient Ice* region (seasonal sea ice zone) by melt/freeze season onset dates reveals a complex influence of sea ice variations on low cloud LWP seasonality. We find that lower tropospheric stability is the primary factor affecting the seasonality of cloud LWP. Our results suggest that variations in sea ice melt/freeze onset have a significant influence on the seasonality of low‐level cloud LWP by modulating the lower tropospheric thermal structure and not by modifying the surface evaporation rate in late spring and midsummer. We find no significant dependence of the May low‐level cloud LWP peak on the melt/freeze onset dates, whereas and September/October low‐level cloud LWP maximum shifts later in the season for earlier melt/later freeze onset regions. The Arctic low cloud LWP seasonality is controlled by several surface‐atmosphere interaction processes; the importance of each varies seasonally due to the thermodynamic properties of sea ice. Our results demonstrate that when analyzing Arctic cloud‐sea ice interactions, a seasonal perspective is critical.

## Key Points

- The Arctic low cloud liquid water path seasonal cycle exhibits a surface‐type dependence
- Sea ice affects liquid water path seasonality by modulating lower tropospheric stability
- Regions of earlier sea ice melt and later freeze onset show a larger cloud liquid water path variance

## 1 Introduction

A factor making the Arctic region (poleward of 60°N) the most sensitive region to climate change is the large coverage of sea ice and its pronounced seasonal changes (Blunden & Arndt, 2016; Lindsay et al., 2009; Maslanik et al., 2007). Arctic sea ice modulates surface albedo, turbulent heat, and momentum fluxes at the ocean‐atmosphere interface (Serreze et al., 2007) and thermodynamic and radiative processes associated with water vapor, clouds, and aerosol feedbacks (see review by Budikova, 2009). As such, Arctic sea ice cover plays an important role in regulating atmosphere‐surface energy exchanges at high latitudes with effects on the large‐scale atmospheric circulation (Overland & Wang, 2010; Taylor, Hegyi, et al., 2018; Wu et al., 2014; Wu & Zhang, 2010; Zhang et al., 2008). Various parameters have been used to quantify sea ice variability from different perspectives, including the sea ice extent, concentration, thickness, and age (Kwok & Untersteiner, 2011; Maslanik et al., 2007; Stroeve et al., 2012), as well as the melt season timing and length (Mortin et al., 2016; Perovich et al., 2007; Perovich & Polashenski, 2012; Stroeve et al., 2014). The timing of melt onset and its duration are thought to impact the freeze season and subsequent winter sea ice thickness and volume anomalies (Bitz et al., 1996; Laxon et al., 2003). Among the dynamic and thermodynamic processes responsible for Arctic sea ice variability, cloud‐sea ice interactions are one of the most poorly understood aspects and the focus of several recent studies (Kay et al., 2016; Letterly et al., 2016; Liu & Key, 2014; Morrison et al., 2018; Taylor et al., 2015). A better understanding of sea ice‐cloud interactions provides insights into the Arctic climate and may ultimately improve climate simulations.

It is widely accepted that changes in cloud cover and cloud properties play a pivotal role in sea ice growth and melt by modulating Arctic surface radiative and turbulent fluxes (Maykut & Untersteiner, 1971; Morrison et al., 2012; Persson, 2012; Persson et al., 2017). Cloud radiative effects depend on the season, atmospheric thermodynamic state, and cloud properties, such as cloud phase, cloud optical depth, and cloud base height (Chen et al., 2006; Shupe & Intrieri, 2004; Sun & Shine, 1994). Considering the annual cycle, Arctic clouds warm the surface via longwave cloud radiative effects most of the year except for a few months in summer when shortwave cloud radiative effects dominate (Boeke & Taylor, 2016; Intrieri et al., 2002; Kay & L'Ecuyer, 2013; Morrison et al., 2012; Schweiger & Key, 1994; Wang & Key, 2003, 2005). Low‐level clouds, with bases below 3 km (Chernykh et al., 2001; Taylor et al., 2015), have a stronger influence on the Arctic surface energy budget than higher clouds due to their frequent occurrence and proximity to the surface and because they are often composed of supercooled liquid (e.g., Kay et al., 2008, 2012; Kay & Gettelman, 2009; Shupe & Intrieri, 2004; Winton, 2006). The strong cloud radiative effect makes the low‐level liquid clouds an important driver of changes in the surface temperature and the growth and melt of sea ice (Kay et al., 2008; Liu & Key, 2014; Mortin et al., 2016; Serreze & Barry, 2011; Wu & Lee, 2012; Zhang et al., 1996).

Recent studies point to the important influence of sea ice on Arctic low‐level cloud formation, based on results derived from surface observations (Eastman & Warren, 2010; Sato et al., 2012), remotely sensed data (Kay et al., 2011; Kay & Gettelman, 2009; Liu et al., 2012; Palm et al., 2010; Schweiger et al., 2008; Taylor et al., 2015; Wu & Lee, 2012), and observationally constrained models (Barton & Veron, 2012; Cuzzone & Vavrus, 2011; Kay et al., 2011; Schweiger et al., 2008; Vavrus et al., 2011). Sato et al. (2012) reported that Arctic open water areas tend to have a larger amount of clouds than sea ice‐covered areas. Model simulations also suggest that low‐level clouds occur more frequently and have a larger liquid water path (LWP) during low sea ice cover years (Barton & Veron, 2012). The long‐term decreasing trend of sea ice is expected to be associated with more low‐level clouds (Wu & Lee, 2012) and enhanced Arctic amplification (e.g., Boeke & Taylor, 2018; Holland & Bitz, 2003; Screen & Simmonds, 2010; Serreze & Barry, 2011; Vavrus et al., 2009; Yoshimori et al., 2014). The total variance (excluding annual cycle and linear trend) in cloud cover from July to November may also be related to sea ice‐cloud interactions (Liu et al., 2012).

Several studies have attempted to explain how sea ice affects cloud amount. One of the direct atmospheric responses to Arctic sea ice loss is an increased lower‐tropospheric heating and moisture content by surface turbulent fluxes (Boisvert et al., 2015; Boisvert & Stroeve, 2015; Francis et al., 2009; Overland & Wang, 2010; Taylor, Boeke, et al., 2018). The enhanced turbulent heat fluxes from the surface and subsequent horizontal and vertical convergence of moisture and energy support the formation of Arctic clouds (Curry et al., 1995, 1996). Indirectly, a reduction of sea ice cover during fall and winter reduces lower tropospheric static stability (LTS) and deepens the planetary boundary layer, favoring cloud formation (Barton et al., 2012; Francis et al., 2009; Jun et al., 2016; Pavelsky et al., 2011; Taylor et al., 2015).

Many of the previous Arctic sea ice‐cloud interaction studies focused on interannual or longer time scales. It remains an open question to what extent the coupling mechanisms of Arctic sea ice and low‐level clouds at interannual scales are relevant at the seasonal scale. This study explores the influence of Arctic sea ice on low‐level clouds at the seasonal time scale. The seasonal variation of Arctic low‐level clouds is determined by many interrelated factors, including incoming solar radiation, sea ice coverage, sea ice thickness, atmospheric thermodynamic and dynamics, and cloud microphysics. In this study, we use the surface‐type stratification as in Morrison et al. (2018) to investigate the influence of surface type and the seasonal variation of sea ice on the seasonality of low‐level Arctic clouds.

The remainder of this paper is organized as follows. The data are presented in section 2. Section 3 shows seasonal variations of sea ice coverage as well as their onset dates of melt and freeze seasons; while in section 4, we present seasonal variations of low‐level cloud LWP over different surface types. Section 5 examines the key factors that determine seasonal variations of low‐level cloud LWP. A brief discussion of other cloud properties including ice water path (IWP) and total water path is provided in section 6. A summary of our findings is presented in section 7.

## 2 Data

Instantaneous cloud property and colocated sea ice concentration (SIC) data for the period from July 2006 through June 2010 are obtained from the Ed RalB1 CALIPSO‐CloudSat‐Clouds and the Earth's Radiant Energy System (CERES)‐Moderate Resolution Imaging Spectrometer (MODIS) (C3M) data set (Kato et al., 2010). C3M provides column‐integrated and vertical profiles of cloud properties such as cloud fraction, liquid water content (LWC), and ice water content (IWC) from merged satellite radar‐lidar‐imager retrievals averaged over CERES footprints (~20 km). C3M also includes SIC retrievals from the Special Sensor Microwave/Imager for 2006–2007 and Special Sensor Microwave Imager/Sounder for 2008 to 2010 (Cavalieri et al., 1996). CERES and SIC footprints are mapped together by convolving the overlapping portions of the footprints using the CERES point spread function (Kato & Loeb, 2005).

Cloud liquid and ice water amounts within C3M are derived from a combination of passive retrievals from MODIS radiances using the CERES science team Edition 3 retrieval algorithms (Minnis, Sun‐Mack, Chen, et al., 2011; Minnis, Sun‐Mack, Young, et al., 2011) and cloud boundary retrievals from CALIPSO and CloudSat. CloudSat‐derived IWC and LWC are converted to the extinction coefficient using the relationship given by Fu (1996) for ice and by Minnis et al. (1998) for liquid. Note that in Fu (1996), it is assumed that ice crystals are hexagonal. Particle size is required for the conversion from CloudSat radar reflectivity to IWC. If CloudSat particle size is not available, MODIS‐derived particle size is used. The extinction coefficient integrated over all cloud layers in the column is normalized by the optical thickness derived by MODIS (Kato et al., 2011). Cloud phase discrimination is based on CloudSat‐derived LWC and IWC profiles partitioned using the temperature profile. CloudSat LWC is 25% smaller than the limited in situ measurements in the Arctic (Austin et al., 2009). Uncertainty in C3M cloud fraction is small (~0.01) especially when clouds are thin, as CALIPSO is extremely sensitive to cloud water and ice (e.g., Avery et al., 2012). However, comparisons with surface‐based remote sensing observations indicate a 25–40% under detection of clouds below 500 m (Liu et al., 2017) from combined CloudSAT‐CALIPSO retrievals. Uncertainties in Arctic air and surface temperature are considered to be ±2 K (Divakarla et al., 2006; Henderson et al., 2013). The biases in these data are not expected to have a strong surface‐type dependence; our comparisons across surface types and regions are expected to be robust.

On any given day, there are up to 2,759 footprints in the latitude band of 70–82°N. Because these footprints occur over the same locations about every 2 weeks, the temporal resolution of the footprint‐level data is very coarse. To increase the temporal resolution, we grid the footprint‐level data—including SIC, vertical profiles of temperature, water vapor, and cloud properties—by averaging all available data within a grid box. When the spatial resolution is sufficiently coarse, the gridded data have a daily temporal resolution. Our results are obtained at the resolution of 2° in longitude by 0.5° in latitude (~50 × 50 km). This ensures the data at the majority of grid points (65%) has a temporal resolution shorter than 3 days and 95% of grid points have a temporal resolution shorter than a week. We then construct the 4‐year mean (2007–2010) annual cycle at each calendar day from 1 January to 31 December of SIC, water vapor content (WV), cloud LWP, cloud IWP, LTS, and temperature.

The European Centre for Medium‐Range Weather Forecasts Interim Re‐Analysis (ERA‐Interim) data set (Dee et al., 2011) shows significant improvements in the global hydrological cycle in terms of water vapor, clouds, and precipitation over ERA‐40 (Uppala et al., 2008). Therefore, we use the surface evaporation flux from the ERA‐Interim data set for the period 2007–2010, interpolated to a 2°×0.5° longitude‐latitude grid, as the same as the regridded C3M data.

## 3 Surface‐Type Stratification and Sea Ice Melt and Freeze Onset Dates

**Figure 1:**
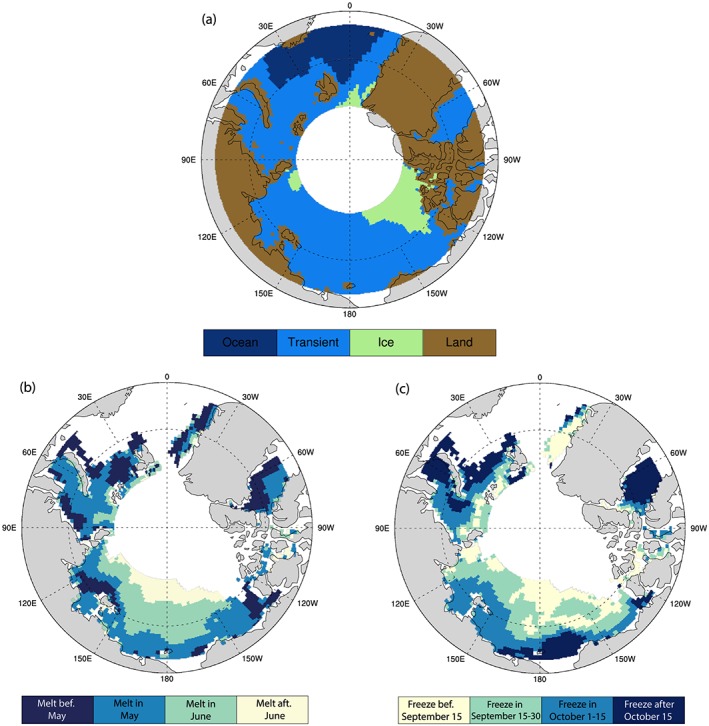
Maps of (a) surface type. (b) and (c) are maps of subregions of the *Transient Ice* regions based on onset dates of (b) sea ice melt season and (c) sea ice freeze season.

We characterize grid points into four types (Figure 1a). (i) A grid is called *Land* if the sum of area percentage of ocean and sea ice in that grid is less than 99% over more than 50% of the time; for the grid points not belonging to *Land* type, (ii) *Permanent Ocean* type is a grid where SIC < 15% over 99% of the time; (iii) *Permanent Ice* type is a grid where the SIC exceeds 15% over 99% of the time; and (iv) the remaining grids are placed into the *Transient Ice* type. Note that the sum of area percentage of ocean and sea ice (i.e., 100% minus land fraction) for a grid box can vary because of the limited sampling. Each 2° × 0.5° grid box is not sampled all of the time, and the exact sampling within the grid box differs. Therefore, in this study, we consider the grid box not completely covered by ocean or sea ice more than a half of time as *Land*, which is consistent with the terrain boundary (see Figure 1a). Most areas within 70–82°N are characterized as the *Transient Ice* type. The *Permanent Ocean* surface type lies mainly over the Barents Sea. *Permanent Ice* type is mainly in the Canadian Archipelago region over the longitude bands from 170°W to 90°W at 82°N, and the longitudinal coverage shrinks with a decrease in latitude until to a very narrow longitude band around 135°W at 75°N.

By definition, only *Transient Ice* grid points possess a pronounced seasonal variation in SIC. Next, we consider spatial patterns of the *onset date of sea ice melt and freeze* to characterize the differences in the seasonal variations of Arctic sea ice coverage and their potential relationship with Arctic low‐level clouds. We define the onset date of sea ice melt at a grid point as the day after which its SIC shows a negative daily tendency for more than 80% of the time with a total decrease of SIC exceeding 15% within a 1‐month period. This condition guarantees a significant change in sea ice cover around the melt onset dates and also eliminates the influence of sea ice regrowth after a short‐lived melting event or due to divergence/convergence of the ice pack. Similarly, the onset date of sea ice freeze is defined as the day after which SIC shows an increasing daily tendency for more than 80% of the time with the total increase of SIC exceeding 15% within a 1‐month period.

Note that the melt and freeze onset dates defined in this study differ slightly from previous studies (e.g., Andreas & Ackley, 1982; Belchansky et al., 2004; Bliss & Anderson, 2014; Collow et al., 2015; Hegyi & Deng, 2017; Huang et al., 2018; Kwok et al., 2003; Lindsay, 1998; Markus et al., 2009; Mortin et al., 2016; Persson, 2012; Rigor et al., 2000; Smith, 1998; Stroeve et al., 2014; Vaughan et al., 2013). Melt onset in most previous studies does not necessarily correspond to a decrease in SIC but rather that the snow pack on top of the sea ice. Comparing the melt and freeze dates used in this study with previous studies shows a general consistency with a delay 1–2 weeks (see supporting information).

**Figure 2:**
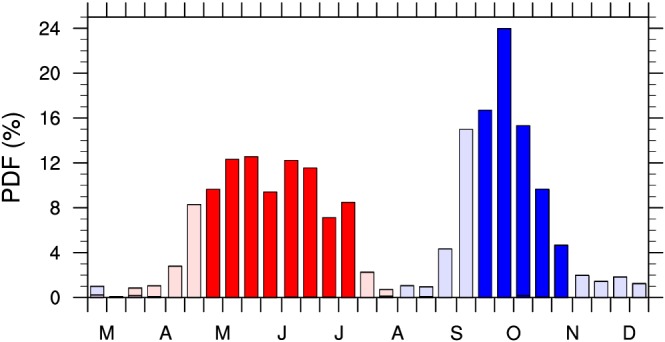
Probability density function (PDF, units: %) of onset dates of sea ice melt season (red and pink bars) and onset dates of sea ice freeze season (blue and light blue bars) over all grid points in *Transient Ice* region. Red and blue bars indicate the PDF within the 10th and the 90th percentile.

We further divide the *Transient Ice* region into four subregions where sea ice melt starts before May, in May, in June, and after June or four subregions where sea ice freezes before 15 September, 15–30 September, 1–15 October, and after 15 October. Figures 1b and 1c indicate the geographic location of those subregions based on onset dates of sea ice melt and freeze. In general, Figure 1 shows that higher latitudes tend to have a later sea ice melt and earlier freeze, consistent with the seasonal cycle of solar insolation. However, there are large zonal deviations in the timing of the onset of sea ice melt and freeze. This indicates that local processes (e.g., ice albedo, water vapor, clouds, and atmospheric transports) influence the total energy input into the surface and the seasonal variations of sea ice (Liu & Schweiger, 2017; Mortin et al., 2016; Persson, 2012). The onset dates of sea ice melt range from April to July (Figure 2). The grid points with melt season onset dates earlier than May (dark blue shadings in Figure 1b) are located in the rims of *Permanent Ocean* and the coastal regions. The grid points with a sea ice melt onset date after June (yellow shadings in Figure 1b) are located at higher latitudes and mainly in the longitude band 120°E to 150°W. The onset dates of sea ice freeze (Figure 1c) shows a narrower range from September to November (Figure 2), and October marks sea ice freeze for most grid points. The color scheme for Figure 1c is deliberately set to be the reverse order of Figure 1b such that the spatial pattern of the onset of sea ice freeze looks similar to that of the melt onset; regions with an earlier melt onset date have a later freeze onset date. The onset date of the sea ice melt is positively correlated with annual mean SIC (Figure S4) (spatial correlation ~ 0.5), while the onset date of the sea ice freeze onset has a large negative correlation with the annual mean SIC (spatial correlation ~ −0.60). This indicates that the late sea ice melt and early freeze of a region influences the annual mean SIC (Mortin et al., 2016; Perovich et al., 2007; Perovich & Polashenski, 2012; Stroeve et al., 2014).

**Figure 3:**
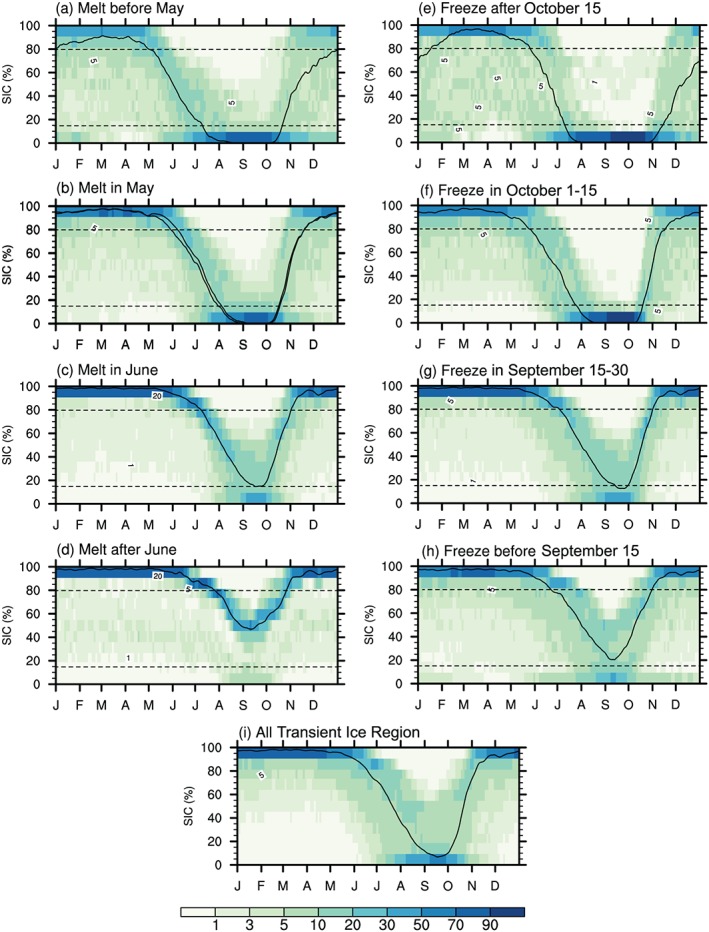
Seasonal cycle of PDF (units: %) of SIC over different regions grouped based on the onset date of sea ice melt (a–d) and those grouped based on the onset date of sea ice freeze (e–h) and over all grid points (i). Black curve indicates the areal‐mean value. Horizontal lines denote the boundary value of SIC between marginal ice zone and the pack ice (80%) and boundary value between open ocean and marginal ice zone (15%) according to U.S. National Ice Center and Parkinson (2014).

We calculated the probability density function (PDF) of 4‐year SIC data over *Transient Ice* grid points that have different sea ice melt and freeze onset dates for each calendar day from 1 January to 31 December. The width of PDF characterizes the spatial variability (degree of dispersion) of the SIC within a target region. Figures 3a–3d show the (4‐year) seasonal variations of the PDF of SIC over the grid points that have different sea ice melt onset dates, Figures 3e–3h are for sea ice freeze onset, and Figure 3i considers all *Transient Ice* grid points. It is seen that regardless of an early or late sea ice melt onset date, most regions have high values of SIC (above 80%) before melt onset and gradually evolve toward a nearly ice‐free state (below 15%) after onset, confirming the effectiveness of our onset date detection methods. It can also been seen that the temporal evolution from sea ice freeze onset to an ice‐covered state is faster (~1–2 months) than that from sea ice melt onset to the minimum sea ice state (~2–3 months), consistent with results shown in Figure 2.

## 4 Seasonal Variations of Low‐Level Cloud LWP Over Different Surface Types

**Figure 4:**
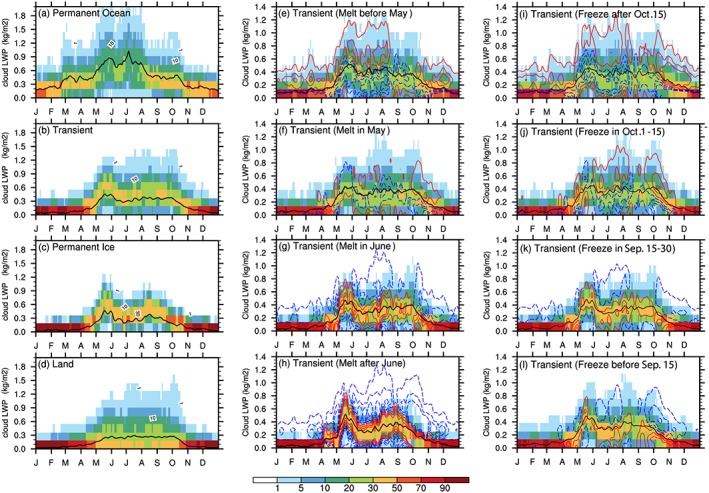
Seasonal cycle of low‐level cloud LWP (units: kg/m^2^) represented by the PDF (shadings) and areal‐mean value (black curve) over four surface types as indicated by Figure 1a: (a) *Permanent Ocean*, (b) *Transient Ice*, (c) *Permanent Ice*, (d) *land*, as well as over four subregions of *Transient Ice* with different (e–h) sea ice melt and (i–l) freeze onset dates as indicated by Figures 1b and 1c. Contours indicate the difference of the PDF for each subregion with the PDF over all grid points belonging to *Transient Ice* type (b), with contour levels at ±2, ±5, and ±8. Positive/negative differences are presented by the red solid/blue dashed contours.

Low‐level clouds dominate the annual cycle of the total Arctic cloud amount (Eastman & Warren, 2010; Taylor et al., 2019). Our analysis focuses on the low‐level cloud LWP because of their strong influence on Arctic surface cloud radiative fluxes (e.g., Shupe & Intrieri, 2004). A brief discussion on the IWP and total water path is provided in section 6. Cloud LWP seasonality is presented as PDFs over the *Transient Ice* region (Figure 4b). We further sort the *Transient Ice* region by sea ice melt and freeze onset dates over this region to investigate the sensitivity of low‐level cloud properties to the seasonality of sea ice. We also show the PDFs of low‐level cloud LWP over *Land*, *Permanent Ocean*, and *Permanent Ice* as references to investigate the sensitivity of cloud LWP to different surface types.

The onset dates of sea ice melt and freeze are strongly related. Therefore, seasonal variations of cloud properties and atmospheric variables over *Transient Ice* with earlier/later sea ice freeze onset can be inferred from the seasonal variations over grid points of *Transient Ice* with later/earlier sea ice melt onset. For this reason, our discussion focuses on the stratification based on melt onset dates unless the results obtained for different freeze onset dates provide additional information.

Table 1 and the left column of Figure 4 show the seasonal variations of low‐level cloud LWP revealing distinct features in both the annual mean and seasonal evolution over different surface types. Specifically, the annual mean low‐level cloud LWP over *Transient Ice* is 0.22 kg/m^2^, smaller than over *Permanent Ocean* (0.46 kg/m^2^) but larger than *Land* (0.15 kg/m^2^) and *Permanent Ice* (0.16 kg/m^2^). In cold months (November through April), low‐level cloud LWP varies little across surface types; *Land*, *Permanent Ice*, and *Transient Ice* surfaces all show a small LWP and weak spatial variability (a narrow width in the PDF). Similar findings have been reported using ground observed and satellite passive radiometer retrieval data (Liu et al., 2012; Vowinckel, 1962). Over *Permanent Ocean*, however, substantial low‐level cloud LWP is found in cold months with the (areal) mean greater than 0.2 kg/m^2^ and a noticeable spatial variability. The temporal evolution of the low‐level cloud LWP over *Permanent Ocean* shows a continuous increase in the first half of the year (January‐June) and a decrease in the remainder of the year. Other surface types exhibit a strong increase in low‐level cloud LWP during warm months (May through September) and a gradual decrease in LWP after September‐October. It is noted that the *Permanent Ice* and *Transient Ice* surfaces show a unique midsummer evolution of LWP, i.e., decreasing from mid‐May to the end of June and then increasing again through mid‐August. This local minimum in the LWP around July is weaker over *Transient Ice* than over *Permanent Ice*.

**Table 1:** Annual Means of the Sea Ice Concentration (SIC), Liquid and Ice Water Content of the Low‐Level Cloud (LWP and IWP) and the Total Water Content (TWP), Water Vapor Below 3 km (WV), Surface Evaporation Rate, Lower Tropospheric Stability (LTS), and Temperature at Surface Level (T_s_) and 700 hPa (T_700hPa_) Level Over the Four Surface Types and the Subregions of *Transient Ice* Region Based on the Melt Season Onset Dates

Variable	Surface type							
	*Permanent ocean*	*Transient ice*					*Permanent ice*	*Land*
		All	Melt before May	Melt in May	Melt in June	Melt after June		
SIC (%)	0.02	59.51	46.0	59.3	73.3	79.6	88.0	3.62
LWP (kg/m^2^)	0.46	0.22	0.25 (0.24)	0.22 (0.22)	0.20 (0.20)	0.18 (0.20)	0.16	0.15
IWP (kg/m^2^)	0.32	0.225	0.225	0.218	0.221	0.250	0.25	0.25
TWP (kg/m^2^)	0.78	0.445	0.475	0.438	0.421	0.430	0.41	0.40
WV (cm)	5.95	3.97	4.24	4.12	3.88	3.59	3.30	2.95
Evaporation rate (10^−6^ mm/s)	7.66	1.19	1.93	1.28	0.81	0.63	0.61	0.74
LTS (K)	12.95	21.23	19.65	21.21	21.88	22.48	24.09	18.05
T_s_ (°C)	4.45	−9.84	−7.36	−9.51	−10.85	−12.59	−14.41	−17.68
T_700hPa_ (°C)	−13.47	−17.16	−16.46	−16.83	−17.53	−18.39	−18.49	−17.71

*Note*. For LWP, we also provide the annual mean averaged over regions that freeze after 15 October, during 1–15 October, 15–30 September, and before 15 September, in parentheses placed from left to right.

**Figure 5:**
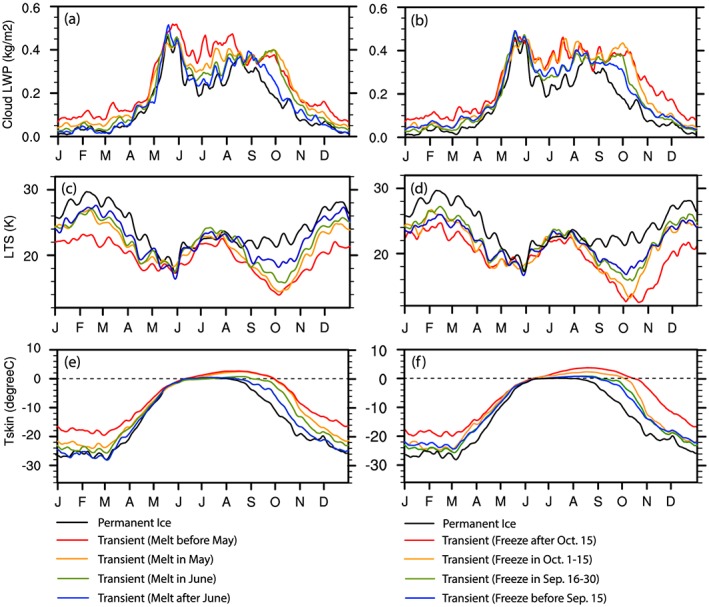
The areal‐mean values of (a,b) low‐level cloud LWP below 3 km (units: kg/m^2^), (c,d) lower tropospheric stability (LTS, units: K), and (e,f) skin temperature (units: K) over *Permanent Ice* and *Transient Ice* regions of different melt and freeze onset date ranges.

We next examine the change in cloud LWP seasonality across *Transient Ice* regions with different sea ice melt (Figures 4e–4h) and freeze onset dates (Figures 4i–4l). The contours in Figures 4e–4l represent differences in the LWP PDF between *Transient Ice* points with different sea ice melt/freeze onset dates and all *Transient Ice* grid points. Positive differences (red contours) indicate a higher likelihood of the specific LWP values relative to the LWP PDF of all *Transient Ice* grid points (Figure 4b) and vice versa for negative difference (blue contours). In Figures 5a–b, we further gather the areal mean seasonal variation of LWP over *Transient Ice* with different onset date ranges and the *Permanent Ice* surface type to make it easier to compare. We found several key results that indicate a complex relationship between the sea ice variation and seasonal cycle of the low‐level cloud LWP as listed below.

1. The May low‐level cloud LWP peak does not shift earlier/later over earlier/later sea ice melt regions as would be expected if the reduction in sea ice was contributing to cloud LWP seasonality.
2. Alternatively, the autumn LWP decrease shifts to an earlier/later time over regions where sea ice freeze earlier/later, clearly evident in October‐November. This indicates a sea ice influence on the cloud LWP seasonality in Autumn.
3. Earlier/later sea ice melt regions possess larger/smaller low‐level cloud LWP particularly in midsummer and a broader/narrower PDF, contributing to a less clear local minimum in the LWP around July over earlier sea ice melt regions. Later/earlier sea ice freeze regions tend to possess larger/smaller low‐level cloud LWP and a broader/narrower PDF particularly in October and November. This also suggests a sea ice influence on the magnitude as well as spatial variability of LWP.

## 5 Physical Processes Linking Sea Ice to the Seasonal Variations of Low‐Level LWP Over Different Surface Types

According to previous studies (Chen et al., 2011; Francis et al., 2009; Jun et al., 2016; Overland & Wang, 2010; Pavelsky et al., 2011; Taylor et al., 2015), sea ice impacts on the Arctic low‐level cloud formation by modulating local evaporation and affecting static stability in the lower troposphere. Therefore, we investigate the annual cycle of lower tropospheric WV (below 3 km) and LTS over different surface types to address the following questions: Why is the annual mean and seasonal cycle of the low‐level cloud LWP dependent on surface type? What factors explain the shape of the LWP seasonal cycle over *Transient Ice* and the regional differences across sea ice melt onset dates? What factors (besides sea ice variation) play a dominant role in the seasonal cycle of LWP? Are these factors modified by the nature of underlying surface?

### 5.1 Moisture Condition

The annual mean of lower tropospheric WV varies by surface type. Lower tropospheric WV shifts from larger to smaller values across surface types from *Permanent Ocean*, *Transient Ice*, *Permanent Ice*, to *Land* (Table 1). Among the *Transient Ice* regions with different melt season onset dates, annual mean WV is larger over earlier melt regions. This is consistent with the dependence of annual mean LWP on the surface type (Table 1); therefore, regional differences in lower tropospheric WV may contribute to the annual mean difference of cloud LWP to surface type. The dependence of the annual mean WV on the underlying surface could result from changes in the local evaporation rate. The annual, areal‐averaged surface evaporation rate from ERA‐Interim shows a similar dependence on the surface type as WV (Table 1). Concurrent with the increased evaporation are increased surface and air temperature that also affect the regional differences in the annual mean WV.

**Figure 6:**
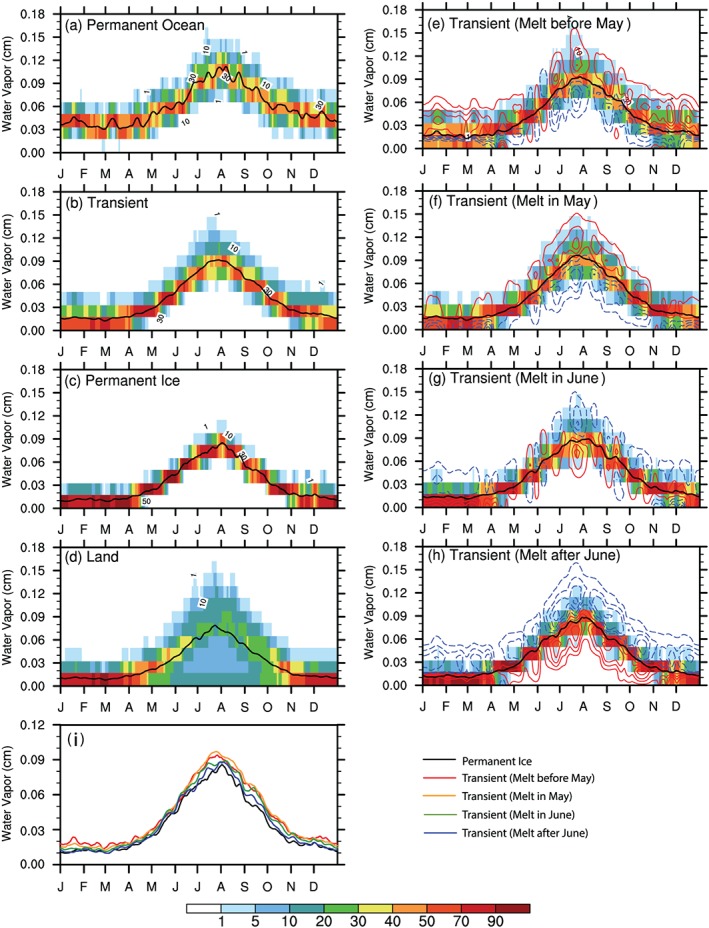
Seasonal cycle of lower tropospheric water vapor (units: cm) represented by the PDF (shadings) and areal‐mean value (black curve) over four surface types as well as over (e–h) four subregions of *Transient Ice* with different melt onset dates. Results over four subregions of *Transient Ice* with different sea ice freeze onset dates are similar, thus not shown. (i) is the areal‐mean values over *Permanent Ice* and *Transient Ice* regions with different melt onset date ranges.

**Figure 7:**
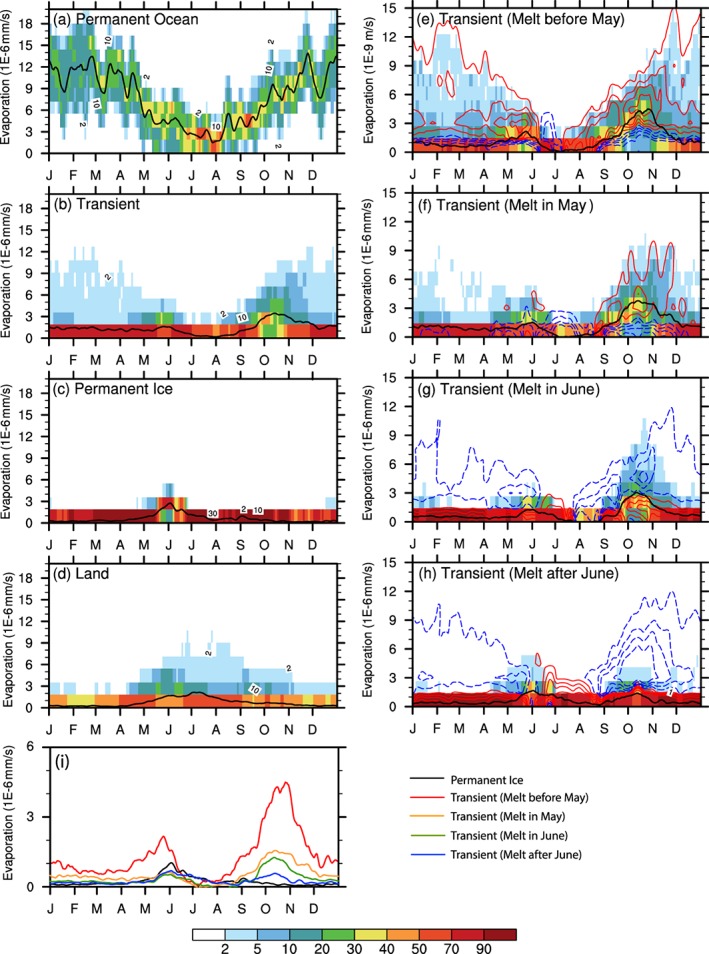
The same as Figure 6 but for the surface evaporation rate (units: 10^–6^ mm/s) from ERA Interim Reanalysis.

The seasonal variation of lower tropospheric WV (Figure 6) indicates smaller values during cold months over the frozen surfaces (including *Land*, *Permanent Ice*, and *Transient Ice* surfaces) yet substantial moisture above *Permanent Ocean* (about 3 cm). *Permanent Ocean* exhibits the strongest evaporation rate in winter, whereas the other surface types have an evaporation rate close‐to‐zero (Figure 7). Seasonally, the evolution of lower tropospheric WV shows an increase from April, reaching a single maximum value around late July and early August, and then decreases afterward for all surface types. Considering *Transient Ice* regions with different sea ice melt onset dates (Figures 6e–6h and 7e–7h), regions with earlier melt have stronger evaporation in cold months and an earlier increase in late spring and early summer. In the late summer and autumn, increased evaporation rate results from the increased temperature contrast between air and water surface (shown in the section 5.2) and is stronger and lasts longer in earlier melt or later freeze regions. Importantly, however, a comparison between Figures 7e–7h and the temporal change of lower tropospheric WV (Figures 6e–6h) indicates that the contribution from local evaporation to the seasonal variation of WV is likely small because their seasonality does not match. Therefore, the surface type and sea ice cover variations do not influence the seasonality of LWP by directly modifying surface evaporation rate.

**Figure 8:**
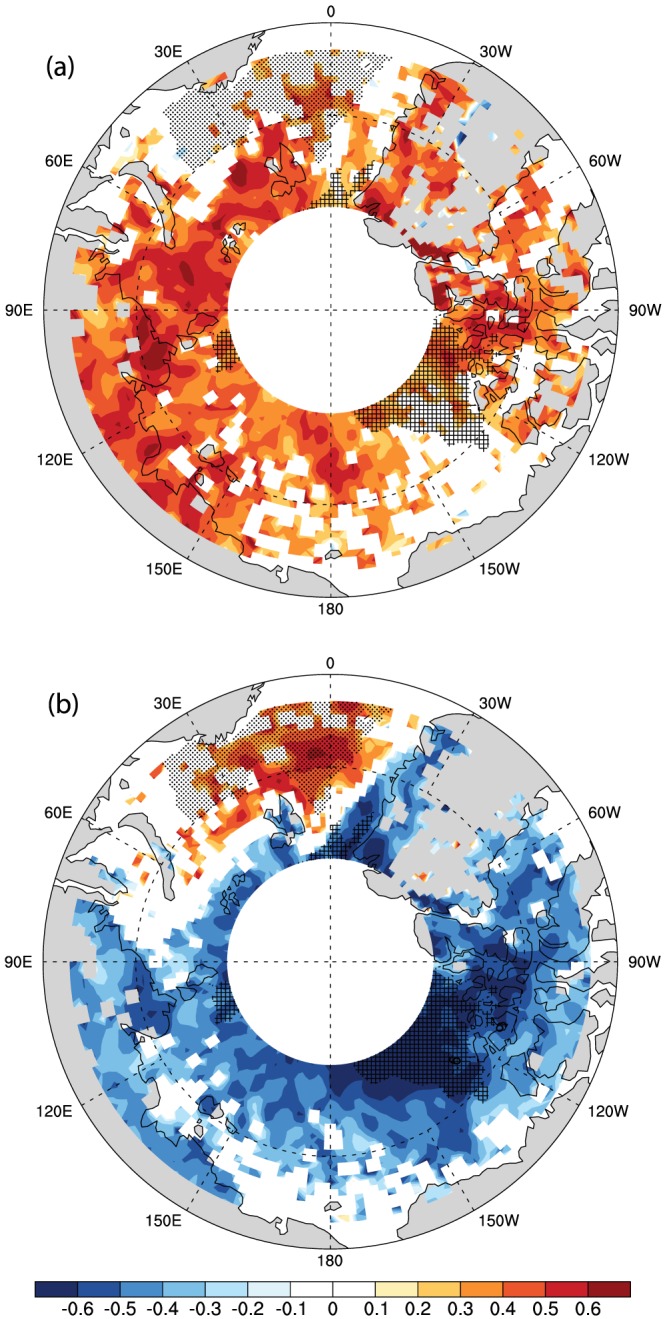
Maps of temporal correlations of low‐level cloud LWP with (a) the total water vapor below 3 km and (b) the lower tropospheric static stability (LTS) during the warm months from April to October. Only correlations above 90% confidence level are shown.

Although we find an annual mean relationship between LWP and lower tropospheric WV across surface types, the relationship is not obvious at the seasonal scale. Comparing Figures 4 and 5 and Figure 6, we can find that both the LWP and lower tropospheric WV evolve together before April and after September. Additionally, the low‐level cloud LWP and lower tropospheric WV in warm months (April to October) are positively correlated over the entire Arctic (Figure 8a) thanks to the increasing of both WV and LWP in the early Spring and their decreasing in the later Autumn. However, the low‐level cloud LWP seasonal cycle shape is inconsistent with the seasonality of lower tropospheric WV over *Permanent Ice* and *Transient Ice* regions from May to September.

### 5.2 Atmospheric Thermodynamic Properties

Seasonal variations in atmospheric thermodynamic properties such as LTS affect cloud formation and evolution (e.g., Kay & Gettelman, 2009). In this section, we investigate the seasonal variation of LTS, in conjunction with seasonal variations of surface skin temperature (T_s_) and air temperature at 700 hPa (T_700hPa_), over different surface types. LTS is defined as the difference between the potential temperature at 700 hPa and the surface (e.g., Klein, 1997; Klein & Hartmann, 1993; Slingo, 1987).

**Figure 9:**
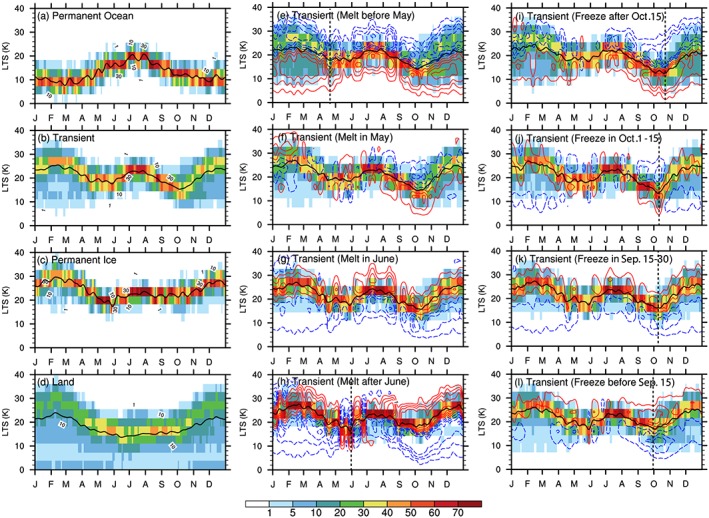
The same as Figure 4 but for the LTS (units: K). The vertical dashed lines roughly mark the timing of the end of decreasing period in the early spring, beginning of rapid increasing period in late summer.

The annual mean, areal‐averaged values of LTS over different surface types (Table 1) indicate that the most unstable condition tends to occur over *Permanent Ocean*, while *Permanent Ice* region is the most stable. The seasonal variation in LTS over *Permanent Ocean* exhibits a maximum of about 20 K in summer and a minimum below 10 K in winter (Figure 9a), whereas *Land* surfaces show an opposite variation with a maximum LTS above 30 K in winter and a minimum below 20 K in summer (Figure 9d). This is consistent with previous studies on the seasonal cycle of LTS in the Arctic, midlatitude land, and ocean regions (Frierson & Davis, 2011; Pavelsky et al., 2011). The seasonal variation of LTS over *Permanent Ice* (Figure 9c) is similar to that over *Land*, other than a small increase in June and July. Over *Transient Ice*, the seasonal variation of LTS (Figure 9b) resembles *Land* and *Permanent Ice* in cold months (October‐April) and resembles *Permanent Ocean* in warm months (May‐September). These LTS seasonal variations agree well with the annual cycle of LTS over the Beaufort‐Chukchi Seas seasonal ice zone reported by Liu and Schweiger (2017).

**Figure 10:**
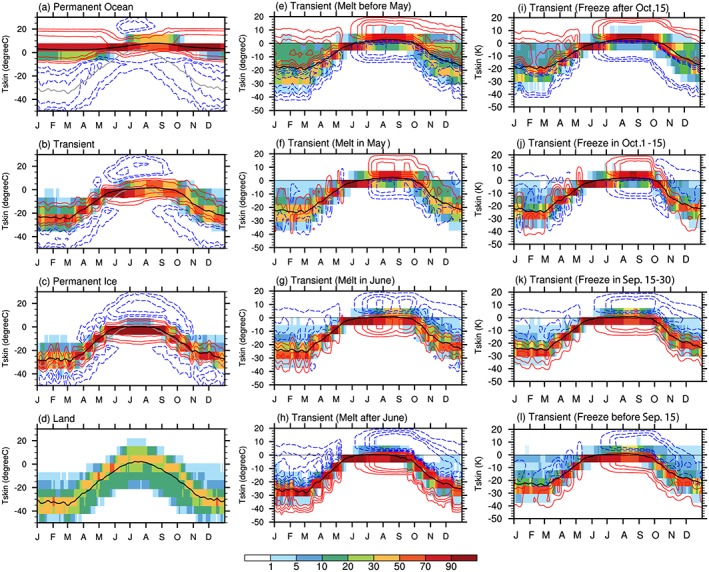
Seasonal cycle of surface temperature (units: °C) represented by the PDF (shadings) and areal‐mean value (black curve) over four surface types, as well as over four subregions of *Transient Ice* with different (e–h) sea ice melt and (i–l) freeze onset dates. Contours in (a)–(c) indicate the difference of the PDF for each type with the PDF over *Land* (d), with contour levels at ±5, ± 10,and ± 20. Gray curves overlaid in (a)–(c) is the areal‐mean surface temperature over *Land* surface. Contours in (e)–(l) indicate the difference of the PDF for each subregion with the PDF over all grid points belonging to *Transient Ice* (b), with contour levels at ±2, ± 5,and ± 8.

**Figure 11:**
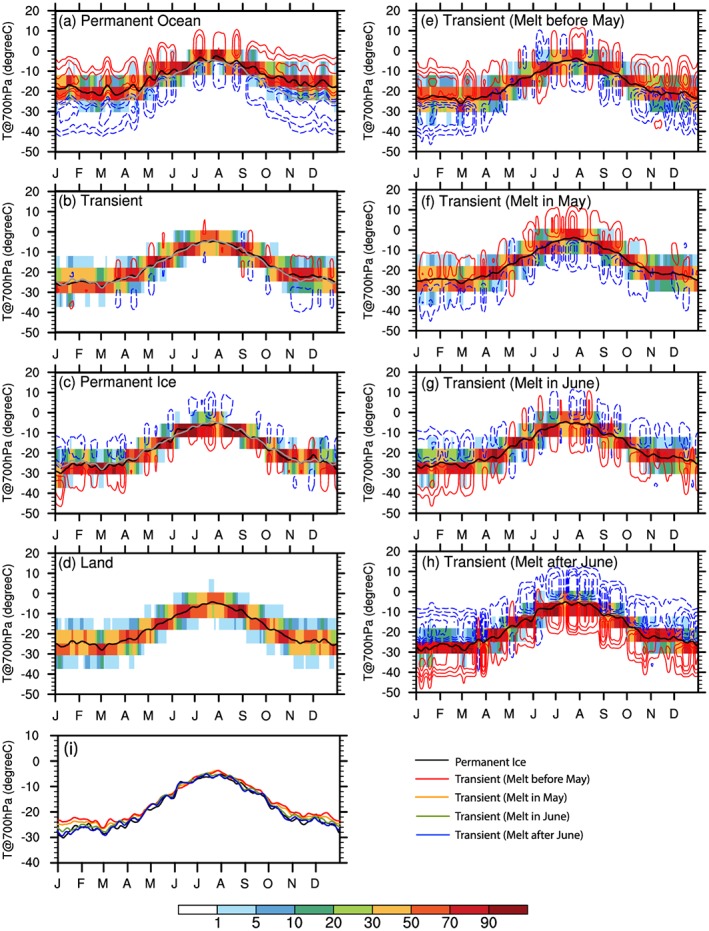
The same as Figure 10, but for the temperature at 700 hPa (units: °C). Results for four subregions of *Transient Ice* with different sea ice freeze onset dates are similar thus not shown. (i) is the areal‐mean values over *Permanent Ice* and *Transient Ice* regions with different melt onset date ranges.

To assess which temperature, surface skin or atmospheric, controls the seasonal variation of LTS over different surface types, we examine the seasonal evolution of T_s_ (Figure 10) and T_700hPa_ (Figure 11). It is expected that the contrast in T_700hPa_ among different surface types is relatively weak because its spatial pattern is mainly determined by atmospheric large‐scale advection. The surface temperature, however, is expected to exhibit a strong dependence on surface type, due to differences in thermal inertia. Sea ice melting and freezing also impact transitions over *Transient Ice* from winter to summer (during melt season) and from fall to winter (during freeze season), making them distinctly different from *Land* or *Permanent Ocean*. In light of the aforementioned discussions, we expect the seasonal variations of LTS over different surface types to be influenced mainly by the thermodynamic properties of the surface.

In cold months, the large thermal inertia and oceanic heat storage keep the *Permanent Ocean* surface above freezing (Figure 10a), while the temperature over the other three surfaces stays from −10 to −40 °C (Figures 10b–10d). Therefore, the leading factor causing the minimum LTS in cold months over *Permanent Ocean* is the large thermal inertia of the ocean, while the smaller thermal inertia of the frozen part of Arctic Ocean and the resulting cold surface skin temperature of the ice/snow covered land is responsible for the maximum LTS in cold months. From spring to midsummer, sea surface temperature warming is weaker than the air, due to large heat capacity of ocean, causing the increase in LTS over *Permanent Ocean* from the spring to midsummer and the LTS peak in summer (Figure 9a). In contrast, frozen and *Land* surfaces with smaller thermal inertia warm more rapidly than the air at 700 hPa in spring, causing a rapid decrease of LTS in early spring (Figures 9b–9d). This stronger surface warming relative to the air above lasts from spring to midsummer over *Land* surface, leading to a minimum LTS in summer (Figure 9d). Surface warming over the *Transient Ice* region (Figures 10b and 10c) becomes slower than the air (Figures 11b and 11c) from late spring to midsummer. The slower surface warming occurs because part of the incoming solar energy is used to melt ice and the large heat capacity of ocean after sea ice melts. These factors explain the increase of LTS from late spring to midsummer over *Transient Ice* regions. From late summer to early autumn when solar radiation decreases, LTS decreases again because the air at 700 hPa cools more rapidly than surface. In autumn, *Transient Ice* regions begin to freeze, and the LTS increases again because of the thermal inertia contrast between an ice covered surface and air. The above also applies to *Permanent Ice* regions although its seasonal variation has a much weaker amplitude.

Among the *Transient Ice* surface type, regions with different sea ice melt onset dates show generally consistent seasonal variations in LTS (Figures 9e–9h), as do seasonal variations of T_s_ (Figures 10e–10h) and T_700hPa_ (Figures 11e–11h). However, there are noticeable differences. First, the earlier melting regions show a larger range in LTS (broader PDF) in wintertime. Second, earlier melting regions tend to be more unstable since they have smaller values of annual mean LTS than later melting regions (Table 1). Third, seen from Figures 9i–9l and 5d, the autumn LTS increase shifts earlier/later over earlier/later freezing region. For example, over the earliest freezing region, the surface starts to freeze before 15 September, transitioning the underlying surface to one with a smaller thermal inertia and causing the surface to cool at a faster rate (Figures 10l and 5f), leading to an earlier LTS increase in Autumn. Comparing Figures 9e and 9h, we further find a slight time shift of the end of the decrease of LTS in the early spring and the beginning of the decrease of LTS in the late summer. Over the earliest melting region, the decrease of LTS in the early spring stops earlier (Figure 9e) because the surface temperature reaches the melting point by late April (Figure 10e) and remains there while the air temperature at 700 hPa (Figure 11e) gradually catches up. The opposite can be said for the later melting regions (Figures 9h, 10h, and 11h). Also, the timing of the rapid decrease of LTS in late summer starts earlier/later over the earliest/latest melting regions. By early July, most sea ice over the earliest melting regions is melted (Figure 3e), and therefore, the surface skin temperature warms (Figure 10e) and drives the LTS decrease after mid‐July (Figure 9e). In contrast, in regions with the latest sea ice melt onset, the surface does not become ice free until August or September, if at all (Figure 4h). As a result, the timing of the increase in the above‐freezing surface temperature (Figure 10h) and decrease in the LTS (Figure 9h) is delayed until mid‐August.

To illustrate how individual surface types and sea ice coverage changes may indirectly influence the low‐level cloud LWP via modifying LTS, we compare the seasonal variation of LTS (Figures 9, 5c and 5d) with that of LWP (Figures 4, 5a, and 5b) over different regions and also examine the spatial pattern of the correlation between low‐level cloud LWP and LTS in warm months from April to October (Figure 8b). Over the *Permanent Ocean* region, where there is abundant water vapor and lower LTS year‐round, the correlation between the seasonal variation of LTS and LWP is positive (Figure 8b). This is because the cloud forming mechanisms are more active in the *Permanent Ocean* region, primarily the north Atlantic Ocean, characterized by frequent large‐scale uplift and warm advection. In this region, a stronger inversion traps more moisture within over marine boundary layer, permitting more low‐level cloud cover (Klein & Hartmann, 1993; Wood & Bretherton, 2006; Wood & Hartmann, 2006; Zhang et al., 2009).

The opposite relationship between LTS and low‐level cloud LWP is found during warm months over *Permanent Ice* and *Transient Ice* regions. The correlation between the seasonal variation of LTS and LWP is negative (Figure 8b), such that a increase in LTS corresponds to a decrease in low‐level cloud LWP (cf. Figures 4c and 4d and Figures 9c and 9d). Over *Permanent Ice* and *Transient Ice* regions, the seasonal minima in LTS (May and October) correspond to the seasonal maxima in LWP. The time shift of the autumn LWP decreases toward an earlier/later time over regions where sea ice freeze earlier/later is consistent with the time shift of autumn LTS increase. The inverse relationship between LTS and LWP over *Permanent Ice* and most of the *Transient Ice* regions and the correspondence between the seasonal LTS minima and LWP maximum suggest that LTS plays an important role in controlling the seasonality of LWP. Low‐level Arctic clouds in *Permanent Ice* and *Transient Ice* regions tend to be decoupled from the surface during the warm season (Shupe et al., 2013) and often have a moisture inversion near cloud top (e.g., Morrison et al., 2012; Shupe et al., 2013; Solomon et al., 2011). Thus, a weaker cloud‐top inversion (weaker LTS) damps turbulence less and allows more moist air from above the cloud to be entrained into the cloud, increasing the cloud LWP. Nevertheless, it is also noteworthy that over earlier melt/later freeze regions, the inverse relationship between LTS and LWP is less significant than later melt/earlier freeze regions and *Permanent Ice* region, suggesting that other factors such as moisture condition may increase the uncertainty in the role of LTS in modulating the seasonal cycle of LWP.

These features over *Permanent Ice and Transient Ice* surfaces, together with the positively correlated LTS‐LWP relationship found over *Permanent Ocean* surface, suggest that the relationship between the temporal evolution of LTS and low‐level cloud LWP during warm months strongly depends on seasonal variations of LTS. Moreover, the seasonal variations in LTS are primarily driven by the seasonality of surface skin temperature. This represents a key mechanism, namely, by modifying the LTS seasonality, through which sea ice influences the seasonality of cloud LWP and its surface type dependence.

### 5.3 Cloud Microphysics

Cloud microphysical processes can also influence the seasonality of low‐level cloud LWP due to temperature‐dependent processes, where colder temperatures promote ice crystal formation and growth (Beesley & Moritz, 1999; Taylor et al., 2019). However, our results suggest that temperature‐dependent cloud microphysics appears to not play a significant role in the differences in cloud LWP seasonality between *Permanent Ice* and *Transient Ice* regions. Figure 11 shows that the 700 hPa temperatures are similar across the different surface types, such that temperature‐dependent aspects of cloud microphysics should not differ between the regions. Additionally, if temperature‐dependent microphysics were a dominant factor to the summer cloud LWP variations, the warmest air temperatures should align with the largest LWP values; this is not the case. Thus, we argue that microphysics are not the primary factor in controlling the surface type dependence of the seasonal variation of low‐level cloud LWP across the Arctic.

## 6 Seasonal Variation of Other Cloud Properties and Their Dependence on Surface Types

**Figure 12:**
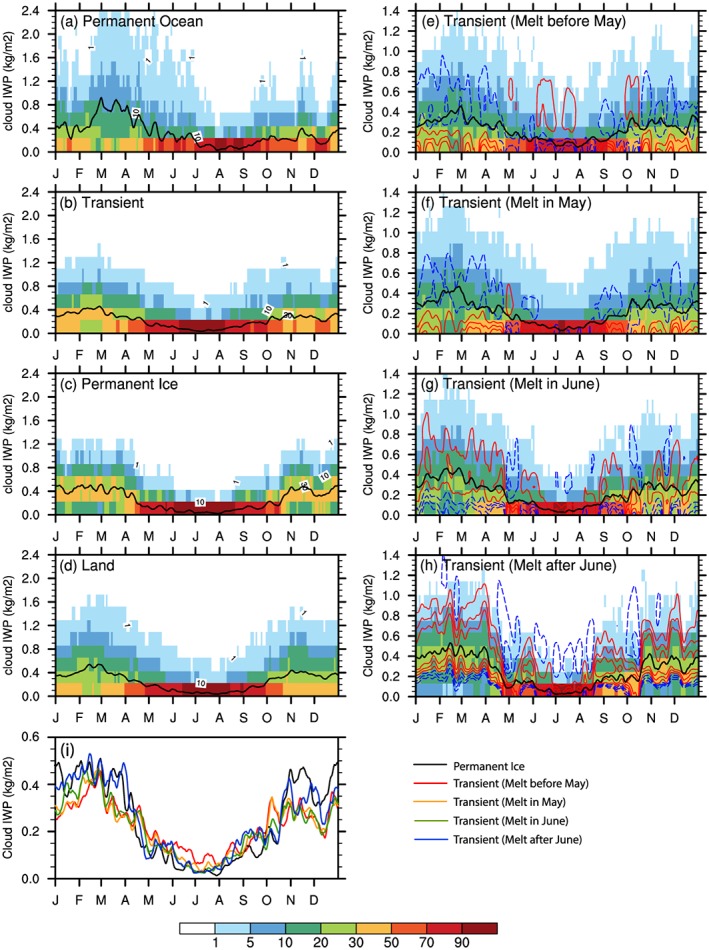
The same as Figure 4 but for the cloud ice water path (IWP) in lower layers below 3 km (units: kg/m^2^). Results for four subregions of *Transient Ice* with different sea ice freeze onset dates are similar thus not shown. (i) is the areal‐mean values over *Permanent Ice* and *Transient Ice* regions with different melt onset date ranges.

We reported the impact of surface type and sea ice melt and freeze onset on the seasonal variation of cloud LWP. We also investigated the seasonal variation of low‐level cloud IWP (Figure 12), which is less dependent on the surface type and mainly follows air temperature. Figures 12e–12h and Table 1 show that earlier/later melting regions tend to have less/more annual mean IWP than the remaining *Transient Ice* region corresponding to seasonal differences in air temperature. The only noticeable difference in the temporal evolution of IWP among the four different surface types is found over *Permanent Ocean* where the seasonal variation of IWP from winter to spring is the strongest. The low‐level total water path follows LWP variations in warm months and IWP in cold months.

## 7 Summary and Conclusion

This study documents the seasonal variation of sea ice, clouds, and the related atmospheric properties in the Arctic region (70–82°N) for the period 2007–2010. We use a surface‐type stratification to investigate the influence of surface type on the seasonality of Arctic low‐level cloud LWP. Four surface types are defined: (i) *Permanent Ocean* where is open water all the year round, (ii) *Land*, (iii) *Permanent Ice* where is covered by sea ice pack all the year round, and (iv) *Transient Ice* where there is a significant seasonal change in sea ice coverage, namely, covered by sea ice in winter but open water in summer. In addition, we further subdivide the *Transient Ice* region into four subregions according to sea ice melt/freeze onset dates to explore the influence of sea ice on the Arctic low cloud LWP.

The annual mean and seasonality of low‐level cloud LWP shows a strong dependence on surface type. Regions with smaller sea ice coverage have a higher surface skin temperature in warm months, stronger evaporation, more lower tropospheric moisture, and weaker LTS; conditions that promote larger low‐level cloud LWP. Regional differences in the annual mean cloud LWP are also affected by other factors, such as air and surface temperature and atmospheric advection from lower latitudes. The seasonal variation of LWP shows a large contrast among different surface types in warm months, whereas in cold months, LWP is close to zero over all surface types except *Permanent Ocean* due to cold temperatures and a lack of water vapor.

The *Permanent Ice* and *Transient Ice* regions exhibit a low‐level cloud LWP seasonal cycle with several distinguishing characteristics: (1) small LWP values during cold months, (2) a primary and secondary peak in May and September/October, and (3) a midsummer local minimum value. Stratifying the *Transient Ice* regions by sea ice melt and freeze onset dates provides several key results: (1) The timing of the May peak in the seasonal variation of low‐level cloud LWP is independent of sea ice melt onset timing; (2) the mid‐summer low‐level areal‐mean cloud LWP values and the width of the PDF are sensitive to melt onset where earlier sea ice melt regions possess larger low‐level cloud LWP with larger spatial variance; and (3) the timing of the autumn LWP peak is sensitive to sea ice freeze onset. Our analysis indicates a complex but close relationship between the sea ice and the seasonality of low‐level cloud LWP.

Variations in LTS is found to be the primary factor controlling the seasonality of cloud LWP and timing of the key turning points of the annual cycle of low‐level cloud LWP. The three primary pieces of evidence include (1) the significant correlation between LTS and LWP across all Arctic regions, (2) the correspondence between the seasonality of LWP and LTS such that the seasonal minima in LTS (May and October) correspond to the seasonal maxima in LWP over *Permanent Ice* and *Transient Ice* region, and (3) variations in LTS explain the warm season differences in the variation of LWP with respect to sea ice melt onset and freeze. Variations in evaporation rate have a smaller contribution than LTS because evaporation rate differences do not contribute to the May LWP maximum and evaporation rate does not explain the midsummer differences in LWP over the regions stratified by sea ice melt onset. Enhanced evaporation rate shows some correspondence to the Autumn LWP maximum in regions except *Permanent Ocean*. However, the evaporation changes linked to variations in underlying surface makes a minor contribution to the seasonal cycle of the water vapor in the lower troposphere. The seasonal structure of the lower tropospheric water vapor shows little contrast by surface type and does not align with the seasonality of low‐level cloud LWP and thus cannot be a primary controlling factor to its evolution.

Despite ruling out the influence of sea ice cover via modifying the surface evaporation, surface characteristics and sea ice melt/freeze onset influence the seasonality of low‐level cloud LWP by modulating the seasonality of LTS and the lower tropospheric thermal structure. The large contrast in the thermal inertia between ocean, sea ice, and land leads to differences in the seasonal variations of surface temperature, especially for the transient ice regions in the transitions between winter to summer (during melt season) and from fall to winter (during freeze season), while the contrast in T_700hPa_ is much weaker.

The primary features in the seasonality of LTS over *Permanent Ice* and *Transient Ice* are explained by the thermodynamic characteristics of the surface and its changes. The May LTS minimum results from the rapid surface skin temperature increase in response to increased solar insolation that results from the low thermal inertia of the ice surface, outpacing the warming in the lower troposphere. The midsummer increase in LTS (which corresponds to a decrease in LWP across *Permanent Ice* and *Transient Ice* regions) occurs because the surface temperature becomes constant at 0 °C due to sea ice melt while the air temperature continues to warm. The LTS decrease from late summer to early autumn results from the rapid cooling of the lower troposphere while the high thermal inertia of the water surface maintains a warmer surface skin temperature. The LTS increase in autumn is closely tied to the surface thermal inertia decrease caused by the sea ice freezing. The timing of the LTS increase is highly dependent on the sea ice variation, which explains the significant time shift of autumn LWP decrease over regions with different sea ice freeze onset dates. Therefore, the seasonality of low‐level cloud LWP follows these variations in LTS and is attributed to the thermodynamic characteristics of the surface.

The influence of sea ice cover on the probability distribution of cloud LWP, to the best of our knowledge, has not been previously demonstrated. We speculate that the apparent enhancement in the variance in low‐level cloud LWP in midsummer may stem from the conditional availability of surface moisture whereby under specific conditions, the cloud layer can access surface moisture resulting in larger LWP values. Additionally, a surface‐type dependent modulation of surface temperature affecting the thermodynamic structure of the lower troposphere may also play a role.

In closing, our results argue that the primary factor controlling the seasonality of low‐level cloud LWP in warm months at different surface types is the variation in lower tropospheric stability controlled by the surface thermodynamic characteristics. The Arctic low cloud LWP seasonality is complex with different processes and atmospheric conditions operating in different months. Taking a seasonal perspective when analyzing Arctic cloud‐sea ice interactions is critical. This refined understanding serves as a useful constraint to assess and improve the representation of the Arctic cloud annual cycle in climate models and increase the fidelity of Arctic climate change projections.

## Supporting information

Supporting Information S1
