# Supplementary material for: Seasonal Variations of Arctic Low‐Level Clouds and Its Linkage to Sea Ice Seasonal Variations
Source: J Geophys Res Atmos. 2019 Nov 21;124(22):12206–26. doi: 10.1029/2019JD031014 (PMC6988461; doi:10.1029/2019JD031014)
Supplement: Supplementary file 1 — Supporting Information S1 [file JGRD-124-12206-s001.docx]

Supporting Information for

**Seasonal variations of Arctic low-level clouds and its linkage to sea ice seasonal variations**

**Yueyue Yu^1^, Patrick C. Taylor^2, *^, Ming Cai^3^**

^1^*Key Laboratory of Meteorological Disaster, Ministry of Education (KLME)/Joint International Research Laboratory of Climate and Environment Change (ILCEC)/Collaborative Innovation Center on Forecast and Evaluation of Meteorological Disasters (CIC-FEMD)/NUIST-UoR International Research Institute, Nanjing University of Information Science and Technology, Nanjing 210044, China*

*^2^ NASA Langley Research Center, Climate Science Branch, Hampton, Virginia, USA*

^3^*Department of Earth, Ocean & Atmospheric Sciences, Florida State University, Tallahassee, Florida, 32304, USA*

**Contents of this file**

Figure S1-S5

Section 1-3

**S1. Comparison of onset dates of melt and freeze based on SIC tendency with previous studies**

Previous studies used surface temperature, brightness temperature, and sea ice concentration from passive microwave satellite data to detect the first sea ice melt and freeze dates (e.g., Smith, 1998; Kwok et al., 2003; Belchansky et al., 2004; Markus et al., 2009; Persson, 2012; Stroeve et al., 2014; Bliss and Anderson, 2014; Collow et al., 2015). Markus et al. (2009) as well as many later studies (Stroeve et al., 2014; Mortin et al., 2016; Hegyi and Deng, 2017; Huang et al., 2018) defined the melt/freeze onset dates based on the sensitivity of microwave brightness temperatures at 19 and 37 GHz to liquid water content in the snow pack. Persson (2012) defined the time when the weekly-mean surface temperature exceeds (falls below) ‑1.15 °C for the first time as the onset of the melt season (freeze season). Other studies used different melting points around -1.15 °C at different stations and different datasets (Andreas & Ackley, 1982; Lindsay, 1998; Rigor et al., 2000). Collow et al. (2015) defined the melt date as the first day after April 1^st^ at which sea ice concentration drops below 15% and after which the sea ice melt persists until the end of summer (called “continuous melt onset date”). This is in accordance with the traditional definition of sea ice extent from the Intergovernmental Panel on Climate Change assessment report (Vaughan et al., 2013).

As we state in the main text, this study is aimed to examine the potential impact from the surface type change to the seasonal variation of low-level clouds, thus the melt onset dates used in this study should involve sea ice coverage changes. Since only when the opening of the water surface or the decreasing of ice-covered surface takes place, significant changes in the water vapor and energy exchange between water and air can be expected. The 'melt onset' in most previous studies does not necessarily mean a decrease in SIC, more so that the snow pack on top of the sea ice begins to melt. The melting of the snow pack would not create a loss in sea ice coverage necessarily. Therefore, our study defines the onset date of melt season at a grid point according to the temporal change of SIC only at a given grid box as introduced in section 3 of the main text.

In this section, we make a preliminary comparison between these different methods of defining onset dates of melt and freeze season. Here, we focus on the comparison with three methods: i) Persson (2012) using specific level of skin temperature, ii) Markus et al. (2009) using brightness temperatures at 19 and 37GHz, and iii) a condition stricter than our definition, which considers the SIC initial threshold (i.e., pack ice, marginal ice zone, or open ocean).

***S1.1 Comparison with onset dates defined following Persson (2012)***

For purposes of demonstration, we display in Figure S2 the seasonal variation of the 4-year mean SIC and skin temperature at five typical grid points. It can be seen that the value of skin temperature when significant melting starts is not a universal constant, instead, it is different at different grid boxes. An example is found at grid box (70°N, 54°E). The remarkable decrease of SIC from 75% to 15% has completed before the skin temperature exceeds -1.15°C. At grid point (80.5°N, 158°E), the timing when skin temperature exceeds -1.15°C turns out to be earlier than our definition. This is because during the period from day 142 to day 184, the SIC value changes very little but changes up and down, not exhibiting a significant and continuous change. At grid point (71°N, 58°E), the melt onset dates derived based on skin temperature threshold and our method are in good agreement. Such a time difference between these two methods may be related to the melting of snow cover over sea ice, local salinity, ice thickness, and other thermodynamic features at specific locations (Fujino et al., 1974; Persson, 2012).

**Figure S1.** Time series of SIC (dashed blue curve for 7-day running mean and solid blue curve for 31-day running mean, left ordinate, unit: %) and skin temperature (red curve, right ordinate, unit: °C) at grid point (a) (80.5°N, 158°E), (b) (75.5°N, 138°W), (c) (71°N, 58°E), (d) (71°N, 170°W), and (e) (70°N, 54°E). The horizontal red line indicates the critical value of skin temperature -1.15°C used by Persson (2012) to detect onset date of melt season. The horizontal blue lines indicate the threshold value of SIC, i.e., 80% and 15%, which are the boundary value of SIC between marginal ice zone and the pack ice and that between open ocean and marginal ice zone, respectively. Green numbers and lines indicate the onset dates of melt (solid) and freeze (dashed) season based on the condition used in this study; purple numbers and lines indicate the onset dates of melt and freeze season using a stricter condition considering the SIC threshold as well; red solid lines indicate the onset dates of melt based on the time when skin temperature increases to above -1.15°C, while red dashed lines indicate the onset dates of freeze based on the time when skin temperature falls below -1.15°C.

***S1.2 Comparison with onset dates derived based on brightness temperature reported by Markus et al. (2009)***

C3M data do not provide brightness temperatures at 19 and 37GHz, therefore, we cannot derive the onset dates following Markus et al. (2009)’s definition. Nevertheless, we compare our sea ice onset dates at two grid points closest to (80.6N, 157.5E) and (75.6N, 136.4W), which are the two points shown in Figures 1-2 of Markus et al. (2009). The 4-year mean SIC time series as well as onset dates at these two grid points are shown in Figs. S1a-b. Note that the time period of the C3M data used in this study is from 2007-2010, thus does not cover year 1998, we can only check qualitatively the correspondence of melt and freeze onset dates between our study and Markus et al. (2009).

Comparison yields that the onset dates of melt and freeze season used in this study correspond to the dates of continuous melt and continuous freeze, rather than dates of the shorter-timescale first melt and freeze event (i.e., early melt and early freeze) in Markus et al. (2009). The feature of temporal evolution of SIC around the onset dates of continuous melt and freeze is overall consistent with that around onset dates by our definition (green lines in Figs. S1a-b). According to the Table 2 of Markus et al. (2009), the average onset dates of continuous melt and freeze are also generally consistent with the value is the corresponding region shown in Figs. 1a-b. Therefore, the sea ice melt and freeze onset dates that we used in this study derived from SIC change are consisten with those in Markus et al. (2009) as well as other studies using the same methods (Mortin et al., 2016; Hegyi and Deng, 2017; Huang et al., 2018) except a possible delay within 1-2 weeks.

This difference in timing of melt onset dates between two methods is probably because the 'melt onset' in Markus et al. (2009) does not necessarily mean a decrease in SIC, more so that the snow pack on top of the sea ice begins to melt. The melting of the snow pack would not immediately correspond to a loss in sea ice coverage. Therefore, our onset dates delayed relative to those using Markus et al. (2009)’s definition.

***S1.3 Comparison with onset dates using a stricter condition considering the SIC threshold***

We also derived a dataset of sea ice onset dates in a stricter sense via adding the threshold of SIC of 80% and 15% as a condition in addition to the SIC tendency condition used in this study. The boundary value of SIC between marginal ice zone and the pack ice is 80%, and that between open ocean and marginal ice zone is 15%. These boundary values are commonly used by operational ice centers (e.g., U.S. National Ice Center) and many previous studies (e.g., Parkinson, 2014). Namely, we define the onset date of melt season at a grid point as the day a) after which its SIC shows a negative daily tendency for more than 80% of the time with a total decrease of SIC exceeding 15% within a one-month period, and c) at which its SIC falls below 80%. And the onset date of the freeze season is defined as the day b) after which its SIC shows an increasing daily tendency for more than 80% of the time with the total increase of SIC exceeding 15% within a one-month period, and d) at which SIC increases to 15% observed after the melt season begins.

Seen from Fig. S1, sea ice melt onset date based on the stricter conditions (purple lines) becomes later only for grid points with a high initial SIC and still above 80% when significant SIC decrease takes place, e.g., at grid point (80.5°N, 158°E), (75.5°N, 138°W) and (71°N, 170°W). Similarly, the freeze onset date becomes later only for grid points with a low SIC in summer and still below 15% when significant SIC increase takes place, e.g., at grid point (80.5°N, 158°E), (75.5°N, 138°W), and (71°N, 58°E). The maps of onset dates of melt and freeze season shown in Fig. S2 show generally consistent pattern with that shown in Figs. 1b-c despite shows a delay at some grid points. Moreover, because we divide the Arctic into four regions based on onset dates at roughly a one-month period, the results derived over earlier/later melting region are consistent. This can be manifested by comparing Figs. S3a-d with Fig. 4e-h.

Adding this condition can make sure the surface nature starts to change significantly, i.e., from packed sea ice region to marginal ice zone, and from open water to marginal ice zone. However, adding this condition will by definition amplify the role of sea ice value itself, instead of its temporal change, on the seasonal variation of cloud properties. For these considerations, we do not include this condition in this study.

**
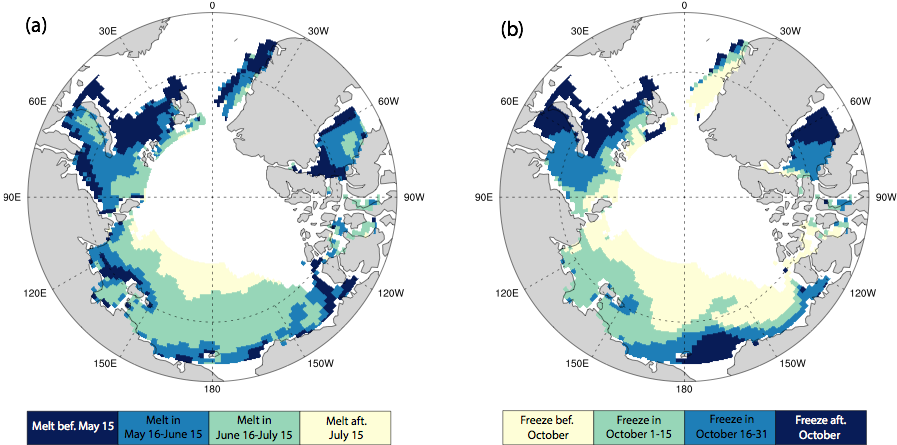
**

**Figure S2.** Maps of sub-regions of Transient Ice regions based on onset dates of (a) melt season and (b) freeze season according to the stricter definition in S1.3.

**Figure S3.** The same as Figs. 3 e-h, but for melt onset dates defined in S1.3.

**S2. Climatological annual mean of sea ice concentration**


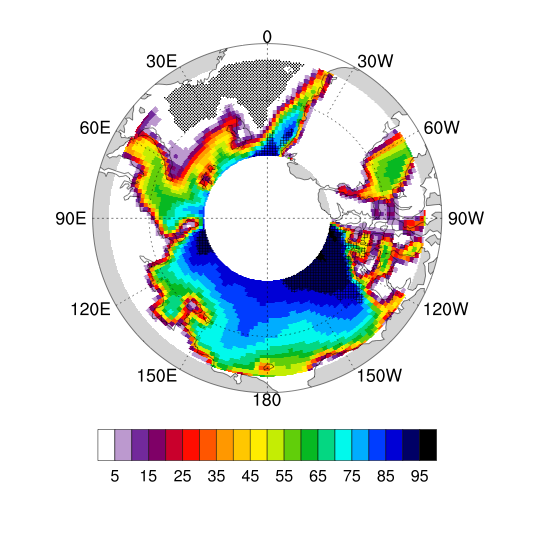


**Figure S4.** Map of climatological annual mean of sea ice concentration (SIC, units: %).

**S3. Supplementary information for relation between evaporation rate and LWP**

It can be seen from Fig. S5 that the evaporation rate shows negative correlations with LWP in warm months from April to October over most areas of Permanent Ocean but positive correlation with LWP over Permanent Ice region and some areas of transient Ice region where sea ice freezes later. But it is obvious that the correlations between LWP and evaporation rate are much less dependent on the surface type and melt/freeze onset dates. In addition, the amplitudes of correlations are overall smaller than those between water vapor below 3 km and LWP and those between LTS and LWP (Fig. 8). This confirms that it is hard for the sea ice change to modify the seasonal variation of LWP via changing water vapor in the lower troposphere by directly changing evaporation rate.

**Figure S5.** Maps of temporal correlations between surface evaporation rate and low-level cloud LWP during the warm months from April to October. Only correlations above 90% confidence level are shown.

**References:**

Andreas, E. L., & Ackley, S. (1982). On the differences in ablation seasons of Arctic and Antarctic sea ice. Journal of Atmospheric Sciences, 39, 440–447.

Belchansky, G. I., Douglas, D. C., & Platonov, N. G. (2004). Duration of the Arctic sea ice melt season: regional and interannual variability, 1979–2001. Journal of Climate, 17, 67–80.

Bliss, A. C., & Anderson, M. R. (2014). Snowmelt onset over Arctic sea ice from passive microwave satellite data: 1979–2012. The Cryosphere, 8, 2089–2100.

Collow, T. W., Wang, W., & Kumar, A. (2015). Prediction of Arctic Sea Ice Melt Date as an Alternative Parameter for Local Sea Ice Forecasting, Science and Technology Infusion Climate Bulletin, 40th NOAA Annual Climate Diagnostics and Prediction Workshop, Denver, CO, USA, 26–29 October 2015, 26–29.

Fujino, K., Lewis, E. L., & Perkin, R. G. (1974). The freezing point of seawater at pressures up to 100 bars, Journal of Geophysical Research, 79, 1792–1797.

Hegyi, B. M., & Deng, Y. (2017). Dynamical and Thermodynamical Impacts of High-and Low-Frequency Atmospheric Eddies on the Initial Melt of Arctic Sea Ice. Journal of Climate, 30, 865–883.

Huang, Y., Dong, X., Xi, B., & Deng, Y. (2018). A survey of the atmospheric physical processes key to the onset of Arctic sea ice melt in spring. Climate Dynamics, 52, 4907–4922.

Kwok, R., Cunningham, G., & Nghiem, S. (2003). A study of the onset of melt over the Arctic Ocean in RADARSAT synthetic aperture radar data. Journal of Geophysical Research, 108(C11), 3363.

Lindsay, R. W. (1998). Temporal variability of the energy balance of thick Arctic pack ice. Journal of Climate, 11, 313–333.

Markus, T., & Stroeve,J. C. (2009). Miller, J. Recent changes in Arctic sea ice melt onset, freezeup, and melt season length. Journal of Geophysical Research, 114, C12.

Mortin, J., Svensson, G., Graversen, R. G., Kapsch, M., Stroeve, J. C., & Boisvert, L. N. (2016). Melt onset over Arctic sea ice controlled by atmospheric moisture transport. Geophysical Research Letter, 43, 6636–6642.

Parkinson, C. L. (2014). Spatially mapped reductions in the length of the Arctic sea ice season. Geophysical Research Letter, 41, 4316–4322.

Persson, P. O. G., Fairall, C. W., Andreas, E. L., Guest, P. S., &. Perovich, D. K. (2002). Measurements near the Atmospheric Surface Flux Group tower at SHEBA: Near-surface conditions and surface energy budget. Journal of Geophysical Research: Oceans, 107, 8045.

Rigor, I. G., Colony, R. L., & Martin, S. (2000). Variations in surface air temperature observations in the Arctic, 1979–1997. Journal of Climate, 13, 896–914.

Smith, D. M. (1998). Observation of perennial Arctic sea ice melt and freeze-up using passive microwave data, Journal of Geophysical Research, 103, 27753–27769. https://doi.org/10.1029/98JC02416

Stroeve, J. C., Markus, T., Boisvert, L., Miller, J., & Barrett, A. (2014). Changes in Arctic melt season and implications for sea ice loss. Geophysical Research Letter, 41, 1216–1225.

Vaughan, D. G., et al. (2013). Observations: cryosphere. In Stocker, TF and 9 others eds Climate change 2013: the physical science basis. Contribution of Working Group I to the Fifth Assessment Report of the Intergovernmental Panel on Climate Change. Cambridge University Press, Cambridge and New York, 317–382.
